# Superior mesenteric artery syndrome: quality of life after laparoscopic duodenojejunostomy

**DOI:** 10.1002/ccr3.1242

**Published:** 2017-12-27

**Authors:** Leonid Barkhatov, Natalia Tyukina, Åsmund A. Fretland, Bård I. Røsok, Airazat M. Kazaryan, Rolf Riis, Bjørn Edwin

**Affiliations:** ^1^ Intervention Centre Oslo University Hospital ‐ Rikshospitalet Oslo Norway; ^2^ Medical Faculty Institute of Clinical Medicine University of Oslo Oslo Norway; ^3^ Medical Faculty Moscow State University of Medicine and Dentistry Moscow Russia; ^4^ Department of Gastrointestinal and Hepatobiliary Surgery Oslo University Hospital ‐ Rikshospitalet Oslo Norway; ^5^ Department of Gastrointestinal Surgery Akershus University Hospital Lørenskog Norway

**Keywords:** Chronic duodenal ileus, laparoscopic duodenojejunostomy, quality of life, superior mesenteric artery syndrome

## Abstract

In this study, we present results after laparoscopic duodenojejunostomy for five patients with protracted superior mesenteric artery syndrome. The procedure can be performed with minimal perioperative risk and very short postoperative stay. It provides significant postoperative symptom relief for many patients with typical presentation of the syndrome.

Superior mesenteric artery syndrome (SMAS) is a rare but complicated medical problem. SMAS, also known as Cast syndrome, chronic duodenal ileus, or Wilkie's syndrome 1, 2, 3, is an intestinal obstruction due to vascular compression of the third part of duodenum by the superior mesenteric artery against the abdominal aorta due to an aortomesenteric angle of <25° 4. Diagnostic work‐up begins with a Doppler ultrasound investigation of abdominal vessels. Typical findings are increased blood flow velocity in the superior mesenteric artery and a reduced aortomesenteric angle 5. Contrast‐enhanced computed tomography and magnetic resonance imaging may be applied for the assessment of the aortomesenteric angle and distance. Arteriographic criteria include a decreased aortomesenteric angle, measuring between 6 and 25° (normal range 38–56°) or the aortomesenteric distance shortened to 2–8 mm (normal range 10–20 mm) 6. As an accompanied symptom, compression of the left renal vein between aorta and SMA was described in the literature as “nutcracker phenomena” 7. Patients with this syndrome may have hematuria and pain in the renal zone.

Upper gastrointestinal contrast series shows dilatation of the stomach and proximal duodenum, delayed gastric emptying of 4–6 h, and obstruction of the horizontal part of duodenum 4, 5, 8.

The disease has typically a chronic intermittent character and mainly affects young women between 10 and 30 years of age 9, 10. Obstruction of the duodenum by SMA can be initiated by any predisposing condition that can lead to loss of mesenteric adipose tissue. SMAS has also been described in patients over 80 years 11, 12.

The SMAS may be seen in association with psychological symptoms. While psychological problems itself can cause pain after food intake, vomiting, appetite loss, and anorexia, the SMAS can also lead to psychological and social problems including depression and anorexia due to the severity of the disease.

Treatment begins with conservative management including nasogastric decompression, correction of electrolyte imbalance, and nutritional optimization. The main goal of conservative treatment was body mass restoration and recovery of water and electrolyte balance.

Duodenojejunostomy for patients with chronic duodenal ileus was first suggested by Bloodgood 13 and performed by Stavely in 1908 14, and since has become a method of choice for this cohort of patients. In 1997, the laparoscopic approach was introduced as an operative management of SMAS 15. Since that, laparoscopic duodenojejunostomy has been described only in few case reports and case series 16, 17, 18, 19, 20.

Other surgical techniques include gastrojejunostomy and lysis of Treitz ligament, both of which have been shown to be inferior to duodenojejunostomy 21, 22.

In this report, we describe a consecutive series of five patients with SMAS who underwent laparoscopic treatment at Oslo University Hospital from January 2007 to June 2013. Patients were referred to the center from local hospitals after ineffective conservative nutritional as well as medical treatment.

The aim of the study was to analyze postoperative outcomes and changes in quality of life after laparoscopic duodenojejunostomy for SMAS.

## Materials and Methods

### Study design and patients’ characteristics

This case series is based on patients that fulfilled the criteria for SMAS and were operated with duodenojejunostomy at Oslo University Hospital between April 2009 and March 2013.

The study includes five female patients with median age of 19 (range 14–33) years. The diagnosis was confirmed with CT angiography for three patients, triplex ultrasound for one patient, and triplex ultrasound combined with CT angiography for the remaining one. The imaging diagnostic shows a narrow aortomesenteric angle (between 10 and 20°).

### Operation technique

**Figure 1:**
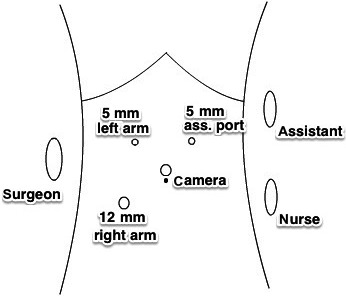
Trocar placement.

A 12‐mm trocar was placed via umbilicus using open‐entry technique. After establishing of pneumoperitoneum, two additional 5‐mm trocars were placed lateral to the rectus abdominis muscle at the right and left side, and 12‐mm trocar below in the right flank (Fig. 1).

**Figure 2:**
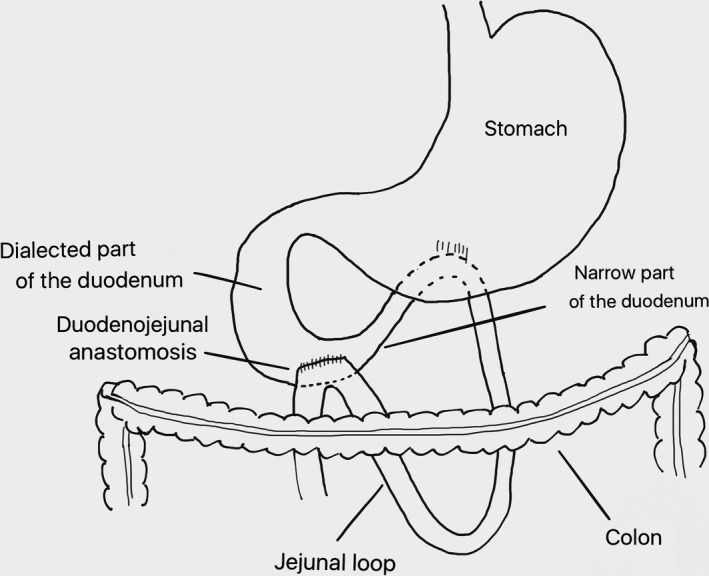
Schematic illustration of duodenojejunostomy.

Duodenojejunostomy was performed as a side‐to‐side anastomosis between the 3rd part of the duodenum and a loop of jejunum approximately 30 cm beyond the ligament of Treitz. The retrocolic anastomosis was performed with a 30‐mm EndoGIA stapling device (Covidien, Mansfield, MA) (Fig. 2). The resulting defect after the insertion of the stapler was closed with a running suture. Patients were allowed feeding ad libitum after the operation.

Intra‐ and postoperative parameters were registered retrospectively from Electronic Health Records retrieved from referring hospitals and from Oslo University Hospital.

To grade intraoperative unfavorable incidents and postoperative complications, the Oslo revision of the Satava approach for surgical error evaluation and the modified Accordion classification were applied, respectively 23.

### Quality of life assessment

The patients were asked to retrospectively assess their preoperative life quality by use of quality of life questionnaires and McGill pain index. The patients performed this during a time period of 6 months to 3 years postoperatively. The same pain index and questionnaires were used to determine their postoperative quality of life during the follow‐up period. The follow‐up period lasted between 1 and 5 years after surgical treatment.

Quality of life assessment was based on the Norwegian version of EORTC QLQ‐C30 version 3.0 and supplemented by EORTC QLQ‐CR29 24. These questionnaires consist of 59 items that correspond to 10 functional scales (“Physical functioning,” “Role functioning,” “Emotional functioning,” etc.), 27 symptoms scales (“Fatigue,” “Nausea and vomiting,” “Pain,” etc.), and Global health status. Answers in each scale were linearly transformed to scores from 0 to 100 points. Higher scores in functional scales and health status indicate better functionality, whereas lower scores in symptoms scales indicate lower expression of symptoms 25.

### Pain scores

As the abdominal pain was determined as the most disabling symptom, the Norwegian short version of McGill pain index (NSF‐MPQ) 26, 27 was used to obtain more detailed assessment of pain. This questionnaire contains a total of 15 characterizing descriptions of pain (four affective and 11 sensory), which are rated on an intensity scale from 0 to 3. In addition, the Present Pain Intensity (PPI) index of the standard MPQ and a visual analogue scale (VAS) were presented.

### Statistical analysis

Post‐ and preoperative scores were tested for normality using the Shapiro–Wilk test, and differences were then compared using pair‐sampled *t*‐test with an alpha level of 0.05. Statistical analysis was performed using the statistical package IBM SPSS version 22.0 (SPSS, Chicago, IL).

### Ethical approval

The study was approved by the Regional Committee for Medical Research Ethics. All patients received written information on the purpose and the design of the study and gave their expressed written consent.

## Results

All patients underwent laparoscopic duodenojejunostomy without conversion to open or hand‐assisted procedure. No intraoperative unfavorable incidents were reported. Median operative time was 95 (range 91–110) min. No measurable bleeding beyond the suction tube (considered to contain <50 mL) was detected.

The postoperative course for every patient was uneventful, and the patients started to drink and eat during the day of the surgery. The median postoperative hospital stay was 1 (range 1–2) day.

Patients were observed during a follow‐up period of 1–5 years.

Postoperative median restoration of the weight was 5 (range 0–8) kg, and the corresponding median improvement of BMI index was 1.8 (range 0–2.8).

### Pain scales

After surgery, patients 1, 2, 3, and 4 presented significant improvement of McGill pain index, visual analogue scale of pain, and PPI score compared to baseline. Patient 5 did not show any improvement (Table 1).

**Table 1:** Improvement in pain scales after laparoscopic duodenojejunostomy

	Mean score, baseline	Mean score, after operation	Difference in mean values	Standard deviation	*P*‐value
McGill pain index^a^	29.8	8.0	21.6	12.9	0.020^a^
Visual analogue scale of pain^a^	8.0	2.2	5.8	3.8	0.026^a^
PPI index^a^	4.4	1.2	3.2	2.0	0.025^a^

a Significant results.

### Quality of life

#### QLQ‐C30 – global health status and functional scales

All patients reported an improvement in global health status after operation. Improvement was found significant with *P*‐value 0.003. Patients 1, 2, 3, and 4 reported considerable improvement in “Physical functioning,” “Role functioning,” “Emotional functioning,” and “Social functioning”. “Cognitive functioning” improved only for patient 3 and 4. Patient 5 showed no difference in “Physical,” “Role,” and “Social functioning” and a slight improvement in “Emotional” and “Cognitive functioning”. The improvement was statistically significant for scales “Physical,” “Role,” “Emotional,” and “Social functioning” (Table 2).

**Table 2:** Improvement in mean scores for EORTC QLQ‐C30 after laparoscopic duodenojejunostomy

	Mean score, before op.	Mean score, after op.	Difference in means	Standard deviation	*P*‐value
Global health status^a^	13.3	63.3	50.0	16.7	0.003^a^
Functional scales					
Physical functioning^a^	37.3	84	46.7	34.3	0.038^a^
Role functioning^a^	6.7	70	63.3	36.1	0.017^a^
Emotional functioning^a^	36.7	73.3	36.7	24.0	0.027^a^
Cognitive functioning	63.3	80	16.7	26.4	0.230
Social functioning^a^	13.3	63.3	50.0	37.4	0.040^a^
Symptom scales					
Fatigue	86.7	42.2	44.4	52.7	0.132
Nausea and vomiting^a^	76.7	13.3	63.3	44.7	0.034^a^
Pain^a^	96.7	30.0	66.7	39.1	0.019^a^
Dyspnoea	33.3	13.3	20.0	18.3	0.070
Insomnia	73.3	40.0	33.3	52.7	0.230
Appetite loss^a^	86.7	20.0	66.7	40.1	0.022^a^
Constipation	53.3	20.0	33.3	52.7	0.230
Diarrhea	33.3	33.3	0.0	N/A	N/A
Financial difficulties	46.7	26.7	20.0	29.9	0.180

a Significant results.

#### QLQ‐C30 – symptom scales

Patients 1, 2, 3, and 4 showed considerable improvement in scores “Pain” and “Nausea and vomiting”. Other factors increased or remained the same individually, with an overall inclination toward an insignificant improvement. All symptom scales were at the same level after operation for patient 5. A significant difference was found in the improvement of mean values for scales “Pain,” “Nausea and vomiting,” and “Appetite loss” (Table 2).

#### QLQ‐CR29 – functional scales

The “Body image” scores improved after operation for patient 1, 2, 4, and 5. For patient 3, the score remained maximum level in 100 points. The “Anxiety scale” values improved for patient 2, 3, and 4 and stayed at the same level for patient 1 (66.7 points) and 5 (0 points). Scale “Weight” was improved for all five patients from mean level 33.3–80.0. “Sexual interest” score was assessed for patients 2, 3, and 5 (patient 1 and 4 were virgins at a baseline time). No difference comparing to baseline was found (100 points for patient 3 and 0 points for patient 2 and 5) (Table 3).

**Table 3:** Improvement in mean scores for EORTC QLQ‐CR29 after laparoscopic duodenojejunostomy

	Mean score, before op.	Mean score, after op.	Difference in mean values	Standard deviation	*P*‐value
Functional scales					
Body image^a^	60.0	93.3	33.3	22.2	0.028^a^
Anxiety	26.7	66.7	40.0	43.5	0.109
Weight	33.3	80.0	46.7	50.6	0.108
Sexual interest^b^	33.3	33.3	0	N/A	N/A
Symptom scales					
Abdominal pain^a^	100	33.3	66.7	40.8	0.022^a^
Bloating	93.3	53.3	40.0	43.5	0.109
Dry mouth	40.0	20.0	20.0	29.8	0.208
Hair loss	33.3	13.3	20.0	18.3	0.070
Flatulence	40.0	26.7	13.3	38.0	0.477
Dyspareunia^b^	33.3	6.7	22.2	19.2	0.184

a Significant results.

b Analysis of scales “Sexual interest” and “Dyspareunia” was based on three answers (patient 1 and 4 was virgin at a baseline period).

#### QLQ‐CR29 – symptoms scales

Symptom scales “Urinary frequency,” “Blood and mucus in stool,” “Stool frequency,” “Urinary incontinence,” “Dysuria,” “Buttock pain,” “Taste,” “Faecal incontinence,” and “Sore skin” scored under 17 points at a baseline (corresponds to answer “I have never felt the symptom”) and were excluded from further analysis. The scale “Dyspareunia” was not applicable.

Scales “Abdominal pain” and “Bloating” improved for patients 1, 2, 3, and 4 and stayed at the same level for patient 5. A significant difference was found in mean values for the scale “Abdominal pain” (improvement from 100.0–33.3, *P*‐value 0.022).

Scales “Dry mouth” and “Hair loss” were improved insignificantly (Table 3).

Parameters “Global health status,” “Physical functioning,” “Emotional functioning,” “Social functioning,” “Pain,” “Appetite loss”, “Body image,” “Anxiety scale,” “Abdominal pain,” McGill pain scale, VAS scale, and PPI index had normal distribution of the samples.

## Discussion

This case series shows that laparoscopic duodenojejunostomy as a treatment of SMAS for patients with severe symptoms, and a complicated medical history may have a successful outcome. Surgery provides relief of symptoms, weight restoration and improves quality of life.

The study shows significant improvement in suitable quality of life indexes and pain scores. However, the results must be regarded with caution, taking into account the small sample size and the fact that patients in this study belong to a selected group with severe symptoms and comprehensive anamnesis. Also, the retrospective nature of preoperative quality of life assessment can cause bias. However, due to the chronic and relatively stable nature of their illness, we believe that patients have a realistic assessment of their preoperative quality of life.

An important aspect of the preoperative investigation is to differentiate patients with SMAS from patients with functional abdominal pain and psychological disorders such as anorexia and others.

Conservative treatment as an initial therapy for SMAS includes nasogastric decompression and total parenteral nutrition, suggested for as long as 7 days in some reports 28. Enteral feedings via nasointestinal tube can also assist in restoring mesenteric fat 29. Medical treatment pursues correction of the fluid and electrolyte balance, a positive nitrogen balance and an increase in body weight, promoting restoration of the retroperitoneal fat tissue with consequent increase in the aortomesenteric angle. Positioning the patient in a knee‐to‐chest or prone position after eating has also been prescribed to improve symptoms, as these maneuvers cause the SMA to be displaced anteriorly, thus increasing the aortomesenteric angle 30, 31.

Because SMAS is a rare condition, no randomized controlled trials have been conducted to compare operative procedures. Historically, a gastrojejunostomy was used to treat SMAS. Gastrojejunostomy has been shown to provide adequate gastric decompression but fails to completely resolve duodenal obstruction leading to a persistence of symptoms necessitating duodenojejunostomy in some cases 21. Persisting obstruction may lead to blind loop syndrome, gastric bile reflux, and ulceration. Strong first described the division of the ligament of Treitz with mobilization of the transverse and ascending duodenum for caudal displacement of the duodenum 22. The disadvantages are that the procedure may be made more difficult or impossible due to adhesions and that caudal displacement of the duodenum cannot always be achieved because of interference with short vessels from the inferior pancreaticoduodenal artery to the duodenum 32.

Recently, several reports of laparoscopic duodenojejunostomy as a treatment for SMAS have been published, reflecting a remarkable progress in laparoscopic techniques during the last two decades 18, 28, 33, 34, 35. Previous open surgery was not considered a contraindication to laparoscopic approach in our series.

This study shows favorable intra‐ and postoperative outcomes without any intraoperative unfavorable incidents or postoperative complications with a median postoperative hospital stay of 1 day.

Laparoscopic procedures are in general associated with faster recovery, less trauma, and shorter hospital stay than open surgery. A survey of the largest series of open duodenojejunostomy carried out between the years of 2002 and 2007 reported an average postoperative stay of 10 days (7–14 days) 36. It is in clear contrast with the median 1 day of postoperative stay in our laparoscopic series with uneventful intra‐ and postoperative course. Better cosmetic results are also an important benefit of laparoscopic approach, especially taking into account that SMAS is usually affecting young patients who are worried about body image and already may have experienced psychosocial symptoms caused by the severity of their disease. A recently published case series study supports our results that laparoscopic duodenojejunostomy is a safe and effective method for the treatment of SMA syndrome 37, 38. Most of the previously published articles on the subject have focused on the feasibility and safety of the procedure, whereas our work focuses upon the quality of life of the concerned patients and thus hopefully provides a new outlook of the subject.

With laparoscopic duodenojejunostomy, the SMAS can be managed with minimal trauma, short hospital and convalescent period, together with good intra‐ and postoperative results. This might question whether laparoscopic treatment might be a viable substitution to the prolonged nasointestinal nutrition for the patients with severe symptoms affecting daily activity and with clean radiological criteria. However, to answer this question, a comparison of two treatment options with large sample size and evaluation of complications is necessary.

## Conclusion

To our knowledge, this is the first study to address quality of life in patients treated by laparoscopic duodenojejunostomy for SMAS. Because SMAS is a rare gastrointestinal syndrome, it is difficult to assess a large cohort of patients. However, even for a case series of five patients, a significant overall improvement of health status, physical activity, emotional, and social status, as well as relief of the most bothering and severe symptoms was achieved.

During the individual analysis of each patient, four of five patients reported complete relief of the symptoms after operation and prolonged follow‐up. The last patient was suffering from chronic pain after previous esophageal surgery, and full pain relief could probably not be expected.

In addition to symptom relief and a great improvement in quality of life, this series demonstrates the benefits of minimally invasive surgery, including minimal pain, short hospital stay, early convalescence and return to work, and good cosmetic outcomes.

## Authorship

LB: participated in study's design, carried out data acquisition, performed the statistical analysis, and drafted the manuscript. NT: helped to draft the manuscript, participated in study's design, and interpreted the data. ÅAF, BIR, AMK, and RR: critically revised the manuscript and implemented important changes in the context. BE: conceived of the study, helped to draft the manuscript, and participated in its design and coordination. All authors read and approved the final manuscript.

## Conflict of Interest

The authors declare that they have no competing interests.

## References

[1] Guthrie Jr, R. H. . 1971 Wilkie's syndrome. Ann. Surg. 173:290–293. 5100100 10.1097/00000658-197102000-00017 PMC1397632

[2] Kwan, E. , H. Lau , and F. Lee . 2004 Wilkie's syndrome. Surgery 135:225–227. 14739859 10.1016/s0039-6060(03)00367-2

[3] Wilkie, D. P. D. 1921 Chronic duodenal ileus. Br. J. Surg. 9:204–214.

[4] Ylinen, P. , J. Kinnunen , and K. Hockerstedt . 1989 Superior mesenteric artery syndrome. A follow‐up study of 16 operated patients. J. Clin. Gastroenterol. 11:386–391. 2760427

[5] Lippl, F. , C. Hannig , W. Weiss , H.D. Allescher , M. Classen , and M. Kurjak 2002 Superior mesenteric artery syndrome: diagnosis and treatment from the gastroenterologist's view. J. Gastroenterol. 37:640–643. 12203080 10.1007/s005350200101

[6] Gustafsson, L. , A. Falk , P. J. Lukes , and R. Gamklou . 1984 Diagnosis and treatment of superior mesenteric artery syndrome. Br. J. Surg. 71:499–501. 6733420 10.1002/bjs.1800710706

[7] Kurklinsky, A. K. , and T. W. Rooke . 2010 Nutcracker phenomenon and nutcracker syndrome. Mayo Clin. Proc. 85:552–559. 20511485 10.4065/mcp.2009.0586 PMC2878259

[8] Barnes, J. B. , and M. Lee . 1996 Superior mesenteric artery syndrome in an intravenous drug abuser after rapid weight loss. South. Med. J. 89:331–334. 8604467 10.1097/00007611-199603000-00015

[9] Akin Jr, J. T. , S. W. Gray , and J. E. Skandalakis . 1976 Vascular compression of the duodenum: presentation of ten cases and review of the literature. Surgery 79:515–522. 1265660

[10] Barner, H. B. , and C. D. Sherman Jr . 1963 Vascular compression of the duodenum. Int. Abstr. Surg. 117:103–118. 14047737

[11] McCue, J. D. , D. S. Nath , and B. A. Bennett . 2006 Image of the month. Superior mesenteric artery syndrome. Arch. Surg. 141:607–608. 16785363 10.1001/archsurg.141.6.607

[12] Roy, A. , J. J. Gisel , V. Roy , and E. P. Bouras . 2005 Superior mesenteric artery (Wilkie's) syndrome as a result of cardiac cachexia. J. Gen. Intern. Med. 20:C3–C4. 16191148 10.1111/j.1525-1497.2005.0201.x PMC1490234

[13] Bloodgood, J. C. X. 1907 Acute dilatation of the stomach‐gastro‐mesenteric ileus. Ann. Surg. 46:736–762. 17862069 10.1097/00000658-190711000-00011 PMC1414443

[14] Stavely, A. L. 1908 Acute and chronic gastromesenteric ileus with cure in a chronic case by duodenojejunostomy. Bull. Johns Hopkins Hospital 19:252.

[15] Bermas, H. , and M. E. Fenoglio . 2003 Laparoscopic management of superior mesenteric artery syndrome. JSLS 7:151–153. 12856847 PMC3015491

[16] Gersin, K. S. , and B. T. Heniford . 1998 Laparoscopic duodenojejunostomy for treatment of superior mesenteric artery syndrome. JSLS 2:281–284. 9876755 PMC3015298

[17] Jo, J. B. , K. Y. Song , and C. H. Park . 2008 Laparoscopic duodenojejunostomy for superior mesenteric artery syndrome: report of a case. Surg. Laparosc. Endosc. Percutan. Tech. 18:213–215. 18427346 10.1097/SLE.0b013e3181661b36

[18] Kim, I. Y. , N. C. Cho , D. S. Kim , and B. S. Rhoe . 2003 Laparoscopic duodenojejunostomy for management of superior mesenteric artery syndrome: two cases report and a review of the literature. Yonsei Med. J. 44:526–529. 12833593 10.3349/ymj.2003.44.3.526

[19] Magee, G. , B. J. Slater , J. T. Lee , and G. A. Poultsides . 2011 Laparoscopic duodenojejunostomy for superior mesenteric artery syndrome. Dig. Dis. Sci. 56:2528–2531. 21643740 10.1007/s10620-011-1757-0

[20] Munene, G. , M. Knab , and B. Parag . 2010 Laparoscopic duodenojejunostomy for superior mesenteric artery syndrome. Am. Surg. 76:321–324. 20349665

[21] Lee, C. S. , and J. C. Mangla . 1978 Superior mesenteric artery compression syndrome. Am. J. Gastroenterol. 70:141–150. 717365

[22] Strong, E. K. 1958 Mechanics of arteriomesentric duodenal obstruction and direct surgical attack upon etiology. Ann. Surg. 148:725–730. 13595530 10.1097/00000658-195811000-00001 PMC1450911

[23] Kazaryan, A. M. , B. I. Rosok , and B. Edwin . 2013 Morbidity assessment in surgery: refinement proposal based on a concept of perioperative adverse events. ISRN Surg. 2013:625093. 23762627 10.1155/2013/625093 PMC3671541

[24] Aaronson, N. K. , S. Ahmedzai , B. Bergman , M. Bullinger , A. Cull , N. J. Duez , et al. 1993 The European Organization for Research and Treatment of Cancer QLQ‐C30: a quality‐of‐life instrument for use in international clinical trials in oncology. J. Natl Cancer Inst. 85:365–376. 8433390 10.1093/jnci/85.5.365

[25] Fayers, P. M. , N. K. Aaronson , K. Bjordal , M. Groenvold , D. Curran , A. Bottomley , On behalf of the EORTC Quality of Life Group . 2001 The EORTC QLQ‐C30 scoring manual, 3rd ed European Organisation for Research and Treatment of Cancer, Brussels, Belgia.

[26] Ljunggren, A. E. , and L. I. Strand . 2006 The norwegian short‐form McGill Pain Questionnaire (NSF‐MPQ). Descriptors, internal consistency and concurrent validity. Eur. J. Pain 10:234.

[27] Melzack, R. 1987 The short‐form McGill Pain Questionnaire. Pain 30:191–197. 3670870 10.1016/0304-3959(87)91074-8

[28] Richardson, W. S. , and W. J. Surowiec . 2001 Laparoscopic repair of superior mesenteric artery syndrome. Am. J. Surg. 181:377–378. 11438278 10.1016/s0002-9610(01)00571-2

[29] Hines, J. R. , R. M. Gore , and G. H. Ballantyne . 1984 Superior mesenteric artery syndrome. Diagnostic criteria and therapeutic approaches. Am. J. Surg. 148:630–632. 6496852 10.1016/0002-9610(84)90339-8

[30] Baltazar, U. , J. Dunn , C. Floresguerra , L. Schmidt , and W. Browder 2000 Superior mesenteric artery syndrome: an uncommon cause of intestinal obstruction. South. Med. J. 93:606–608. 10881780

[31] Welsch, T. , M. W. Buchler , and P. Kienle . 2007 Recalling superior mesenteric artery syndrome. Dig. Surg. 24:149–156. 17476104 10.1159/000102097

[32] Mansberger Jr, A. R. , J. B. Hearn , R. M. Byers , N. Fleisig , and R. W. Buxton 1968 Vascular compression of the duodenum. Emphasis on accurate diagnosis. Am. J. Surg. 115:89–96. 5634681 10.1016/0002-9610(68)90134-7

[33] Agarwalla, R. , S. Kumar , A. Vinay , and S. Anuradha . 2006 Laparoscopic duodenojejunostomy for superior mesenteric artery syndrome. J. Laparoendosc. Adv. Surg. Tech. A 16:372–373. 16968185 10.1089/lap.2006.16.372

[34] Goitein, D. , D. J. Gagne , P. K. Papasavas , R. Dallal , B. Quebbemann , J. K. Eichinger et al. 2004 Superior mesenteric artery syndrome after laparoscopic Roux‐en‐Y gastric bypass for morbid obesity. Obes. Surg. 14:1008–1011. 15329194 10.1381/0960892041719626

[35] Schroeppel, T. J. , W. S. Chilcote , M. D. Lara , and S. N. Kothari . 2005 Superior mesenteric artery syndrome after laparoscopic Roux‐en‐Y gastric bypass. Surgery 137:383–385. 15746797 10.1016/j.surg.2004.10.006

[36] Merrett, N. D. , R. B. Wilson , P. Cosman , and A. V. Biankin . 2009 Superior mesenteric artery syndrome: diagnosis and treatment strategies. J. Gastrointest. Surg. 13:287–292. 18810558 10.1007/s11605-008-0695-4

[37] Sun, Z. , J. Rodriguez , J. McMichael , R. M. Walsh , S. Chalikonda , R. J. Rosenthal , et al. 2015 Minimally invasive duodenojejunostomy for superior mesenteric artery syndrome: a case series and review of the literature. Surg. Endosc. 29:1137–1144. 25701058 10.1007/s00464-014-3775-4

[38] Pottorf, B. J. , F. A. Husain , H. W. Hollis Jr , and E. Lin . 2014 Laparoscopic management of duodenal obstruction resulting from superior mesenteric artery syndrome. JAMA Surg. 149:1319–1322. 25353279 10.1001/jamasurg.2014.1409

